# Fistula of the Parotid Gland

**Published:** 1891-02

**Authors:** A. L. Benedict

**Affiliations:** Buffalo, N. Y.


### Clinical Report.

### FISTULA OF THE PAROTID GLAND.

BY A. L. BENEDICT, M. D., Buffalo, N. Y.

ON October 27th, A. B., a strong, healthy-looking laborer, presented himself with marked lesions of secondary syphilis, from an exposure three months previously. There was an abscess over the ramus of the inferior maxillary, which had been opened a few days before he consulted the writer, but in which pus had again collected, though the incision—a longitudinal one—had scarcely healed. The original wound was reopened and slightly enlarged, when a considerable quantity of pus was evacuated.

The wound was packed with zinc oxide gauze and covered with a light dressing. The patient was placed upon the following prescription:

| | |
|---|---|
| ℞.—Hydrargyri chlor. corros | .20 |
| Potassii iodidi | 10. |
| Syr. sarsaparillæ co. | 100. |
| Aquam ad | 200. |

S. 5 c. c. P. C. [Teaspoonful after meals.] This medicine was replaced later by pills of mercurous iodide .03, three times daily.

The abscess discharged freely for several days when, the pus abating, it became evident that a salivary fistula remained. The patient stated that there was a sufficient supply of saliva and, on drying the mucous membrane in the vicinity of the orifice of Steno's duct and touching the tongue with vinegar, the side of the mouth became moist. Moreover, the upper portion of the external wound had healed and the fistula was slightly below the line of the duct, (from the lower part of the lobe of the ear to a point midway between the ala of the nose and the corner of the mouth,) and farther back than the main duct usually extends. From physiological and anatomical reasons, therefore, it was decided that the fistula was from the gland substance and was not due to occlusion of the duct.

On December 1st, cocaine was injected and the edges of the old wound freshened by the bistoury and curved scissors. Three small catgut sutures, about one-eighth of an inch apart, were introduced and above and below the ends of the wound, rubber adhesive strips were applied so as to approximate the tissues as much as possible, and to relieve all strain upon the sutures. A figure-eight head and chin bandage was put on to prevent movement of the jaw.

The patient was seen daily, but it was thought best not to remove the sutures as soon as is usually done in facial surgery. On December 4th the knots were cut off short, and on the next day the remnants of the sutures were removed. Thus far all had gone well, but on December 6th a swelling of the gland occurred and saliva oozed from one or two suture-holes and through part of the original wound. A temporary dressing of iodoform ointment on lint was applied, and the patient directed to return the next day, when the distension was found to have nearly disappeared, and there was only slight oozing from the wound and suture-holes. The ointment dressing was therefore continued and the swelling diminished gradually and the oozing correspondingly, although for some days the patient, not understanding directions, persisted in pressing the gland to force out the fluid.

On December 13th, what little distension remained had localized itself at a point nearly over the angle of the jaw and seemed to be pointing, the skin presenting a purplish discoloration. An opening was accordingly made, evacuating several drops of clear fluid, undoubtedly saliva, which showed under the microscope red-blood corpuscles, a moderate number of leucocytes and a few epithelial cells. Three days later this opening, as well as the original one, had entirely healed and the swelling had nearly subsided. Iodine ointment was substituted for the iodoform ointment and, on December 22d, the patient stated that the fistula had remained entirely closed and the swelling was barely noticeable. Meanwhile, the general condition of the patient had been much improved and the pills were withheld for a few days on account of the beginning of mercurialisin.

